# Two novel SHP-1 agonists, SC-43 and SC-78, are more potent than regorafenib in suppressing the in vitro stemness of human colorectal cancer cells

**DOI:** 10.1038/s41420-018-0084-z

**Published:** 2018-08-13

**Authors:** Shin-Yi Chung, Yen-Hsi Chen, Pei-Rong Lin, Ta-Chung Chao, Jung-Chen Su, Chung-Wai Shiau, Yeu Su

**Affiliations:** 1 Institute of Biopharmaceutical Sciences, School of Pharmaceutical Sciences, National Yang-Ming University, TAIPEI, Republic of China; 2 Division of Medical Oncology, Department of Oncology, Taipei Veterans General Hospital, TAIPEI, Republic of China; 3 Faculty of Medicine, School of Medicine, National Yang-Min University, TAIPEI, Republic of China; 4 Faculty of Pharmacy, School of Pharmaceutical Sciences, National Yang-Ming University, TAIPEI, Republic of China

**Keywords:** Cancer, Cancer stem cells

## Abstract

Signal transducer and activator of transcription 3 (STAT3) has been shown to play a critical role in the maintenance of cancer stem cells (CSCs). Hence, the inhibition of STAT3 signaling has been suggested to be a viable therapeutic approach for cancers. Moreover, the efficacy of combinations of chemotherapeutic drugs and napabucasin, a small-molecule STAT3 inhibitor, have been assessed in various clinical trials, including those involving patients with metastatic colorectal cancer (CRC).

Two recently developed small-molecule STAT3 inhibitors, SC-43 and SC-78, which can stimulate SHP-1 to inactivate STAT3, were found to have anti-tumor activity. In this study, the inhibitory effects of SC-43, SC-78, and regorafenib (a reference drug) on cell viability, STAT3 phosphorylation, and various stemness properties [e.g., sphere-forming and soft agar colony-forming abilities, CD133^+^/CD44^+^ (stem cell-like) subpopulations, and the expression of several CSC markers] were examined for both HCT-116 and HT-29 human CRC cells. We found that SC-43 and SC-78 but not regorafenib inhibited constitutive and IL-6-induced STAT3 phosphorylation in HCT-116 and HT-29 cells, respectively. Moreover, SC-43 and SC-78 were more potent than regorafenib in suppressing the stemness properties (except stem cell-like subpopulations) of these cells. As expected, SHP-1 knockdown almost completely abolished the suppressive effects of SC-43 and SC-78 on the sphere formation in both cell lines. Furthermore, SC-43 and SC-78 showed synergistic inhibitory effects with oxaliplatin and/or irinotecan on sphere formation. Overall, our results suggest that SC-43 and SC-78 are potent STAT3 inhibitors that may potentially be used in combination therapy for CRC.

## Introduction

Colorectal cancer (CRC) is a malignancy with high incidence and mortality^1^, and the prognosis of patients with CRC is largely based on how the disease was diagnosed^2,3^. Unfortunately, ~50% of patients with CRC develop metastases, and most of these patients have unresectable tumors and need systemic treatments to prolong their survival^4^. Despite some advances in CRC therapy in recent years, the overall survival rate of patients with this disease has not greatly improved^5^, which is most likely attributed to the presence of CRC stem cells (CRCSCs). Indeed, it has been well documented that CRCSCs display an intrinsic tendency towards chemoresistance and may be responsible for tumor regeneration and relapse after conventional therapy^6–9^. Hence, eliminating cancer stem cells (CSCs) along with the bulk of the tumor either by monotherapy or combination therapy has been proposed to be the most effective treatment approach for cancer patients^10^. As the persistent activation of one or more highly conserved signal transduction pathways involved in development and tissue homeostasis, such as the Notch, Hedgehog (Hh), and Wnt pathways, is frequently observed in CSCs^11^, the development of new treatment strategies targeting these critical pathways for controlling stem cell replication, survival, and differentiation is currently being investigated. In addition to these signaling pathways, signal transducer and activator of transcription 3 (STAT3) is another major oncogenic pathway activated in CRC, which can serve as a therapeutic target for this malignancy^12,13^. Interestingly, the constitutive activation of STAT3 has also been observed in CRCSCs^14^, and STAT3 stimulated by IGF signaling could enhance the self-renewal of CRCSCs by upregulating *NANOG* expression^15^. As expected, these studies also demonstrated that the stemness of CRC cells could be markedly abolished by STAT3 inhibition.

The activation of STAT3 involves the phosphorylation of a critical tyrosine residue (Tyr705) mainly by the Janus kinases (JAKs), followed by homodimerization, translocation to the nucleus, and stimulation of the expression of downstream mediators^16^. Hence, small-molecule STAT3 inhibitors targeting the different steps of STAT3 activation have been developed. For example, SD-1029 and WP1066 could suppress Jak2 activation^17,18^. STA-21, Stattic, and S3I-201 could exert inhibitory effects mainly by preventing STAT3 dimerization^19–21^. On the other hand, LLL12, LY5 (a derivative of LLL12), and napabucasin (BBI608) could inhibit STAT3 phosphorylation^14,22,23^. The anti-tumor activity of napabucasin either by itself or in combination with conventional therapeutics has been demonstrated in several recent clinical trials^24^, strongly suggesting the potential of small-molecule STAT3 inhibitors in suppressing metastasis and preventing relapse in patients with varying cancer types by targeting CSCs.

In addition to inhibiting STAT3 phosphorylation, the removal of the phosphate group from Tyr705 is another method of inactivating this transcription factor^25^. Among various protein tyrosine phosphatases that could dephosphorylate and inactivate STAT3^26–28^, Src homology 2 domain-containing protein tyrosine phosphatase 1 (SHP-1) has been suggested to be the most promising drug target for developing small-molecule STAT3 inhibitors because it can dephosphorylate not only STAT3^29^ but also JAKs^30^. In addition, it can function as a tumor suppressor to inhibit the growth of breast^31^, prostate^32^, and pancreatic cancer cells^33^, and its activity and expression levels can be stimulated by several natural and synthetic compounds including two clinical multikinase inhibitors, sorafenib and regorafenib^34^.

In this study, we extend our previous research to assess the inhibitory effects of regorafenib^35^ and two novel SHP-1 agonists, SC-43^36^ and SC-78^37^, on constitutive and inducible STAT3 phosphorylation in HCT-116 and HT-29 human CRC cells, respectively. Furthermore, their effects on the stemness properties of these cells were investigated. Our results showed that SC-43 and SC-78 were more effective than regorafenib in suppressing the activation of STAT3 in both HCT-116 and HT-29 cells. We also demonstrated that SC-43 and SC-78 were more potent than regorafenib in reducing sphere formation, inducing sphere shrinkage, reducing soft agar colony formation, and inhibiting the expression of several CRCSC markers in the human CRC cells. However, the three drugs decreased the CD133^+^/CD44^+^ subpopulations of both HCT-116 and HT-29 cells with equal potency. Interestingly, SC-43 and irinotecan exhibited synergistic effects in suppressing the sphere formation of both HCT-116 and HT-29 cells. The synergistic effects of SC-78 and oxaliplatin in abolishing the sphere formation of HCT-116 cells were also demonstrated. As expected, SHP-1 silencing markedly abrogated the inhibitory effects of SC-43 and SC-78 on the sphere formation of both human CRC cell lines. Collectively, these results suggest that these two novel SHP-1 agonists are promising for further development as adjuncts for CRC therapy.

## Results

### Active STAT3 levels are higher in drug-resistant human CRC cells

**Fig. 1:**
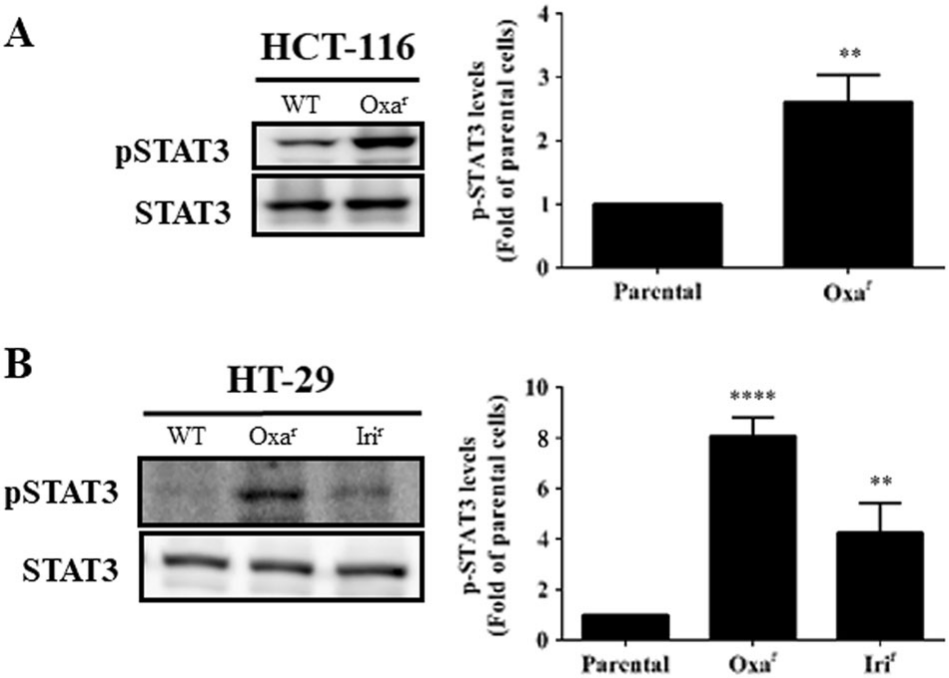
Detection of active STAT3 levels in drug-resistant HCT-116 and HT-29 cells. Total lysates (30 μg) were prepared from **a** parental (WT) and oxaliplatin-resistant HCT-116 cells as well as **b** parental, oxaliplatin-resistant, and irinotecan-resistant HT-29 cells and subjected to western blot analyses using primary antibodies against phospho-specific STAT3 (Tyr705, pSTAT3) and regular STAT3 (loading control). Data (right panels) are the mean ± SD of three independent experiments. *p < 0.05, **p < 0.01, ***p < 0.005, and ****p < 0.001 compared with parental cells by Student’s *t*-test

Higher drug resistance resulting from the activation of the JAK/STAT3 pathway has been reported in various human CSCs^38–40^; thus, we investigated whether this aberrant signaling could also confer drug resistance in human CRC cells. Oxaliplatin-resistant and irinotecan-resistant human CRC cells were established by culturing the HCT-116 and HT-29 cell lines with increasing sublethal concentrations of oxaliplatin and irinotecan until their growth was no longer affected by these drugs. Western blotting was then performed to analyze the active STAT3 levels in these cells. Higher active STAT3 levels were found in both oxaliplatin-resistant HCT-116 and HT-29 cells as well as in irinotecan-resistant HT-29 cells (Fig. 1). These results clearly indicated that STAT3 signaling was abnormally activated in human CRC cells resistant to two first-line anti-CRC drugs.

### Regorafenib, SC-43, and SC-78 are toxic to human CRC cells

Based on our observations, we hypothesized that STAT3 signaling might play a critical role in maintaining and/or enhancing the stemness of human CRC cells and that the suppression of this signaling pathway might reduce the viability of CRCSCs. To investigate whether STAT3 signaling suppression could abolish the stemness properties of CRC cells, the cytotoxic effects of three SHP-1 agonists including regorafenib^35^, SC-43^36^, and SC-78^37^ (their chemical structures are shown in Supplementary Fig. 1) on both HCT-116 and HT-29 cells were evaluated by MTT assays after they were treated with varying doses of the drugs for 48 h. As shown in Table 1, the IC_50_ values of regorafenib, SC-43, and SC-78 were 1.5, 2.9, and 4.2 μM, respectively, for HCT-116 cells, and 3.8, 3.2, and 4.1 μM, respectively, for HT-29 cells.

**Table 1:** Cytotoxic effects of regorafenib, SC-43, and SC-78 on HCT-116 and HT-29 human colorectal cancer cells

Cell line	HCT-116	HT-29
Regorafenib	1.5 ± 0.2	3.8 ± 0.4
SC-43	2.9 ± 0.2	3.2 ± 0.4
SC-78	4.2 ± 0.4	4.1 ± 0.3

Cells (1 × 10^4^/well) were seeded into 96-well plates 1 day before treatment without or with various doses (0.1, 0.25, 0.5, 1, 2, 5, and 10 µM) of regorafenib, SC-43, and SC-78. After 48 h, cell viability was determined by MTT assays, and the IC_50_ values of the three drugs were calculated

### Active STAT3 levels in human CRC cells are markedly reduced by a lower dose of SC-43 and SC-78 but not regorafenib

**Fig. 2:**
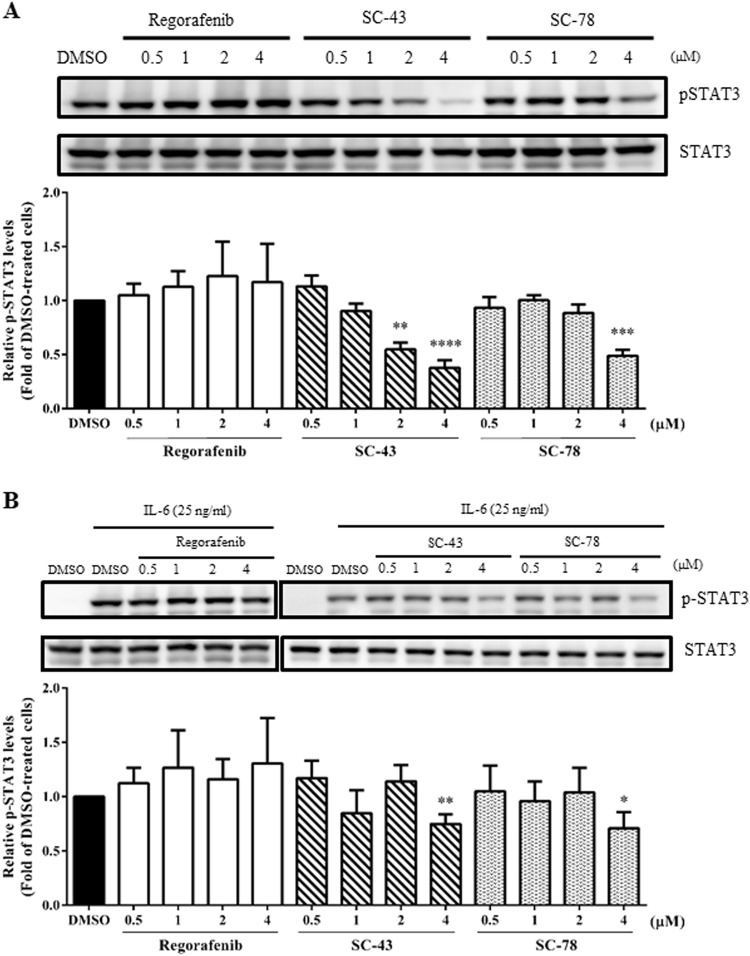
Effects of regorafenib, SC-43, and SC-78 on active STAT3 levels in HCT-116 and HT-29 cells. **a** Total lysates (30 μg) were prepared from HCT-116 cells after treatment with DMSO (vehicle) or different doses (0.5, 1, 2, and 4 µM) of regorafenib, SC-43, and SC-78 for 24 h and subjected to western blot analyses using primary antibodies against pSTAT3 and STAT3. **b** Total lysates were also prepared from HT-29 cells after treatment with DMSO or different doses of regorafenib, SC-43, and SC-78 for 6 h and IL-6 (25 ng/ml) for another 30 min and subjected to western blot analyses using the two primary antibodies. Data (lower panels) are the mean ± SD of three independent experiments. *p < 0.05, **p < 0.01, ***p < 0.005, and ****p < 0.001 compared with DMSO-treated cells by Student’s *t*-test

To further assess whether the three SHP-1 agonists could indeed reduce the levels of active STAT3 in human CRC cells, western blotting was performed using the total lysates prepared from HCT-116 and HT-29 cells after they were treated with the drugs at their respective IC_50_ values. Endogenous levels of active STAT3 in HCT-116 cells were significantly reduced by 2 and 4 μM SC-43 and 4 μM SC-78 but not by 4 μM regorafenib (Fig. 2a). The levels of active STAT3 induced by IL-6 in HT-29 cells were also markedly reduced by 4 μM SC-43 and SC-78 but not by regorafenib (Fig. 2b). Collectively, our results suggest that SC-43 and SC-78 were more effective than regorafenib in lowering active STAT3 levels in both HCT-116 and HT-29 cells.

### SC-43 and SC-78 are more effective than regorafenib in reducing the stemness properties of HCT-116 and HT-29 cells

**Fig. 3:**
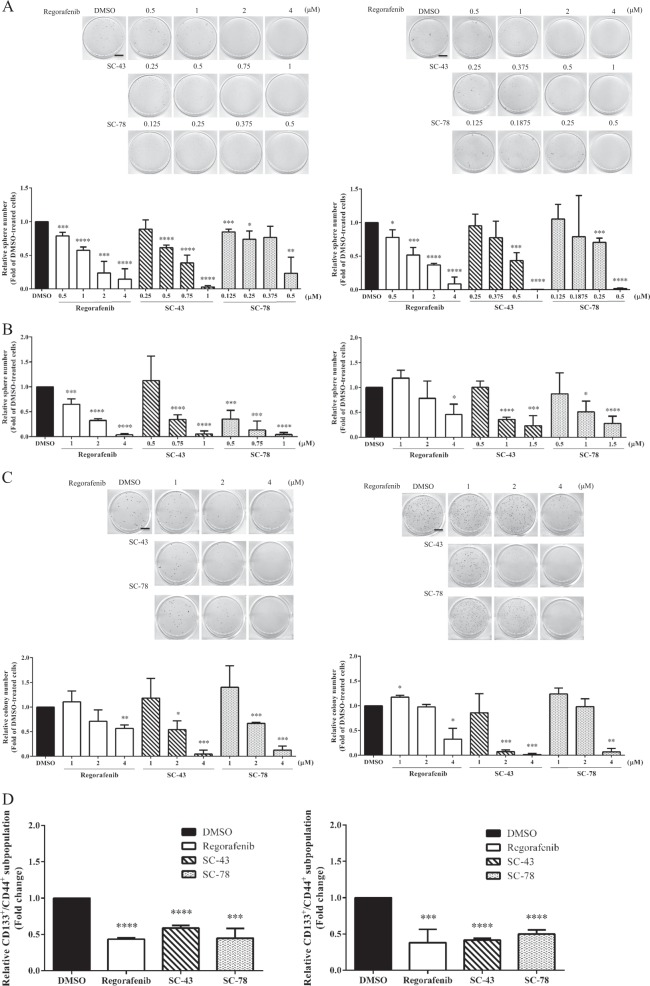
Effects of regorafenib, SC-43, and SC-78 on the stemness properties of HCT-116 and HT-29 cells. **a** HCT-116 (top) and HT-29 (bottom) cells (1 × 10^5^) were cultured in defined media with DMSO (vehicle) or different doses of regorafenib (0.5, 1, 2, and 4 µM), SC-43 (0.25, 0.5, 0.75, and 1 µM for HCT-116; 0.25, 0.375, 0.5, and 1 µM for HT-29), and SC-78 (0.125, 0.25, 0.375, and 0.5 µM for HCT-116; 0.125, 0.188, 0.25, and 0.5 µM for HT-29). After 7 days, spheres stained by MTT were photographed (scale bar = 0.7 cm), and their numbers were quantified by MetaMorph software. Data are the mean ± SD of three independent experiments. **b** HCT-116 (left) and HT-29 (right) cells (1 × 10^5^) cultured in defined media for 5 days were treated with DMSO or different doses of regorafenib (1, 2, and 4 µM), SC-43 (0.5, 0.75, and 1 µM for HCT-116; 0.5, 1, and 1.5 µM for HT-29), and SC-78 (0.5, 0.75, and 1 µM for HCT-116; 0.5, 1, and 1.5 µM for HT-29) for another 7 days. The sphere number was quantified. **c** HCT-116 (top) (1 × 10^3^/well) and HT-29 (bottom) (2 × 10^3^/well) cells were cultured in 0.3% agar containing DMSO (vehicle) or different doses (1, 2, and 4 µM) of regorafenib, SC-43, and SC-78. After 20 days, colonies stained by MTT were photographed (scale bar = 0.7 cm), and their numbers were quantified by MetaMorph software. **d** HCT-116 (left) and HT-29 (right) cells were treated with DMSO or 4 μM of regorafenib, SC-43, and SC-78 for 48 h. The percentage of the CD133^+^/CD44^+^ subpopulations of each sample was determined by flow cytometry after the cells were incubated simultaneously with PE-conjugated anti-CD133 and FITC-conjugated anti-CD44 antibodies. Data are the mean ± SD of three independent experiments. *p < 0.05, **p < 0.01, ***p < 0.005, and ****p < 0.001 compared with DMSO-treated cells by Student’s *t*-test

As active STAT3 has been reported to be essential for regulating the CSCs of a number of cancers including CRC^41^, we next examined the effects of regorafenib, SC-43, and SC-78 on the stemness properties of both HCT-116 and HT-29 cells. Regorafenib, SC-43, and SC-78 inhibited the sphere-forming ability of HCT-116 (IC_50_ values of 1.1, 0.6, and 0.4 μM, respectively) and HT-29 (IC_50_ values of 1.1, 0.5, and 0.2 μM, respectively) cells in a dose-dependent manner (Fig. 3a). To further analyze the toxic effects of regorafenib, SC-43, and SC-78 on pre-formed spheres, the drugs were added 5 days after cells were seeded in the defined media to form spheres. As shown in Fig. 3b, regorafenib, SC-43, and SC-78 were effective in causing the shrinkage of the pre-formed spheres of HCT-116 (IC_50_ values of 1.3, 0.7, and 0.4 μM, respectively) and HT-29 (IC_50_ values of 3.4, 1.0, and 1.0 μM, respectively) cells. In addition, we performed soft agar colony formation assay to examine whether the three drugs could also suppress anchorage-independent growth. As expected, regorafenib, SC-43, and SC-78 inhibited the anchorage-independent growth of HCT-116 (IC_50_ values of 4.4, 2.1, and 2.8 μM, respectively) and HT-29 (IC_50_ values of 3.5, 1.4, and 3.2 μM, respectively) cells (Fig. 3c). Flow cytometry was then performed to analyze the suppressive effects of the three drugs on the CD133^+^/CD44^+^ stem cell-like subpopulations^42,43^ of the human CRC cells. All three drugs effectively reduced the subpopulations of both HCT-116 and HT-29 cells with similar potency (Fig. 3d). Together, our results indicated that SC-43 and SC-78 were more efficient than regorafenib in not only inhibiting the self-renewal and anchorage-independent growth abilities of both HCT-116 and HT-29 cells but also inducing the shrinkage of the spheres formed by these cells.

### SC-43 and SC-78 are more effective than regorafenib in suppressing the expression of stemness markers in HCT-116 and HT-29 cells

**Fig. 4:**
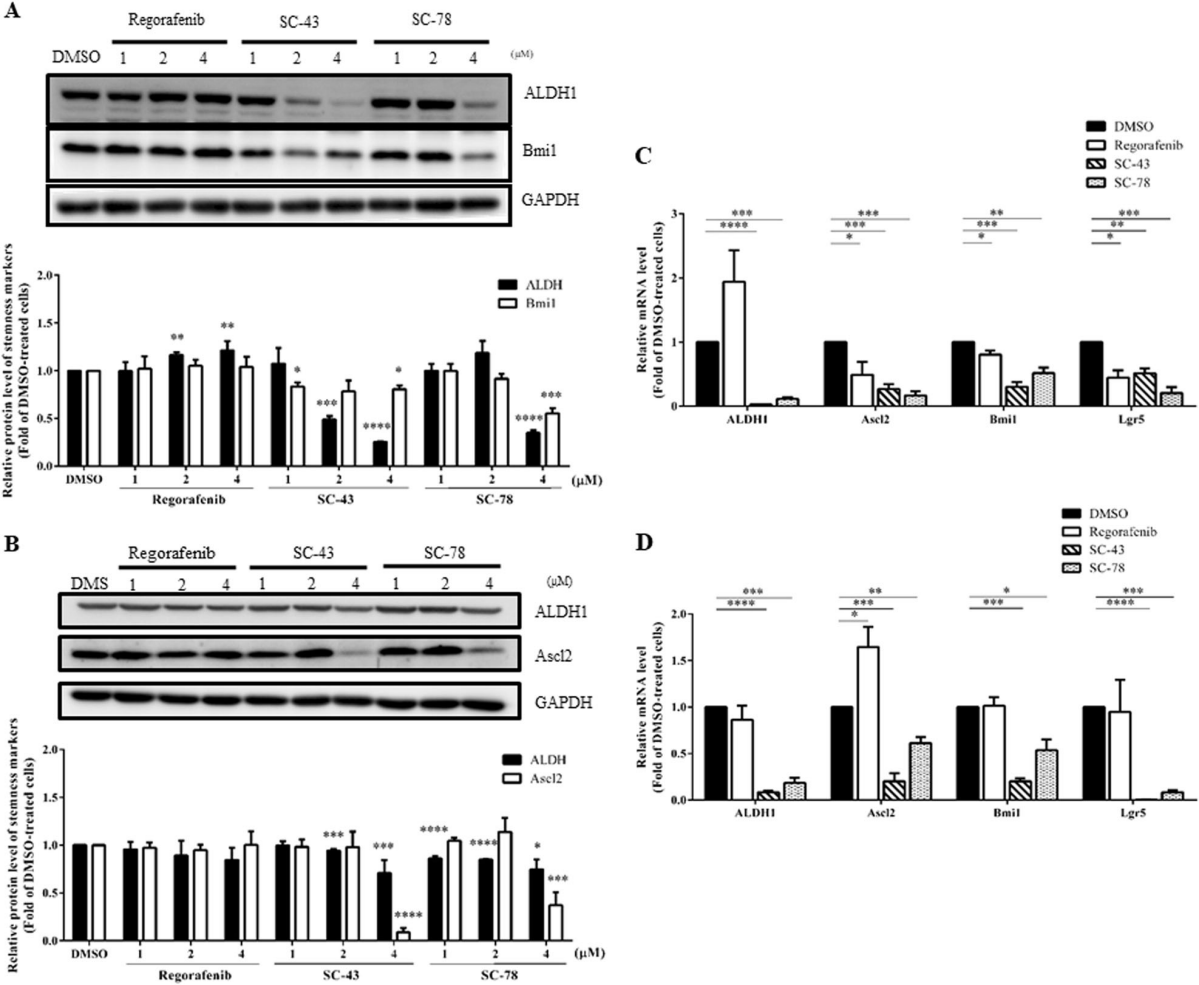
Effects of regorafenib, SC-43, and SC-78 on the expression of stemness markers in both HCT-116 and HT-29 cells. Total lysates (20 μg) were prepared from (**a**) HCT-116 and **b** HT-29 cells after they were treated with DMSO (vehicle) or different doses (1, 2, and 4 µM) of regorafenib, SC-43, and SC-78 for 72 h and subjected to western blot analyses using primary antibodies against ALDH1 and Bmi1 (for HCT-116 cells) or ALDH1 and Ascl2 (for HT-29 cells). GAPDH was used as a loading control. The quantitative results obtained by densitometry are the mean ± SD of three independent experiments (lower panels). *p < 0.05, **p < 0.01, ***p < 0.005, and ****p < 0.001 compared with DMSO-treated cells by Student’s *t*-test. Total RNA samples (5 μg) were isolated from **c** HCT-116 and **d** HT-29 cells after they were treated with DMSO or 4 µM of regorafenib, SC-43, and SC-78 for 48 h and subjected to qRT-PCR analyses to determine the mRNA levels of several CRCSC marker genes. Data are the mean ± SD of three independent experiments. *p < 0.05, **p < 0.01, ***p < 0.005, and ****p < 0.001 compared with DMSO-treated cells by Student’s *t*-test

We examined the effects of the three drugs on the expression levels of several CRCSC markers in both HCT-116 and HT-29 cells by western blot and qRT-PCR analyses. SC-43 and SC-78 but not regorafenib significantly decreased the protein levels of ALDH1 and Bmi1 in HCT-116 cells (Fig. 4a). Similarly, SC-43 and SC-78 but not regorafenib markedly reduced the protein levels of ALDH1 and Ascl2 in HT-29 cells (Fig. 4b). In accordance with these findings, both SC-43 and SC-78 effectively suppressed the mRNA levels of *ALDH1*, *Ascl2*, *Bmi1*, and *Lgr5* in both cell lines (Fig. 4c). However, although the mRNA levels of *Ascl2*, *Bmi1*, and *Lgr5* were significantly reduced in HCT-116 cells after treatment with 4 μM regorafenib, this drug increased the mRNA level of *ALDH1* (Fig. 4c). A similar dose of regorafenib did not affect the mRNA levels of *ALDH1*, *Bmi1*, and *Lgr5* but upregulated the mRNA level of *Ascl2* in HT-29 cells (Fig. 4d). Therefore, these results suggest that SC-43 and SC-78 were more potent than regorafenib in attenuating the stemness of the human CRC cells.

### The inhibitory effects of SC-43 and SC-78 on the sphere-forming ability of HCT-116 and HT-29 cells are abolished by SHP-1 knockdown

**Fig. 5:**
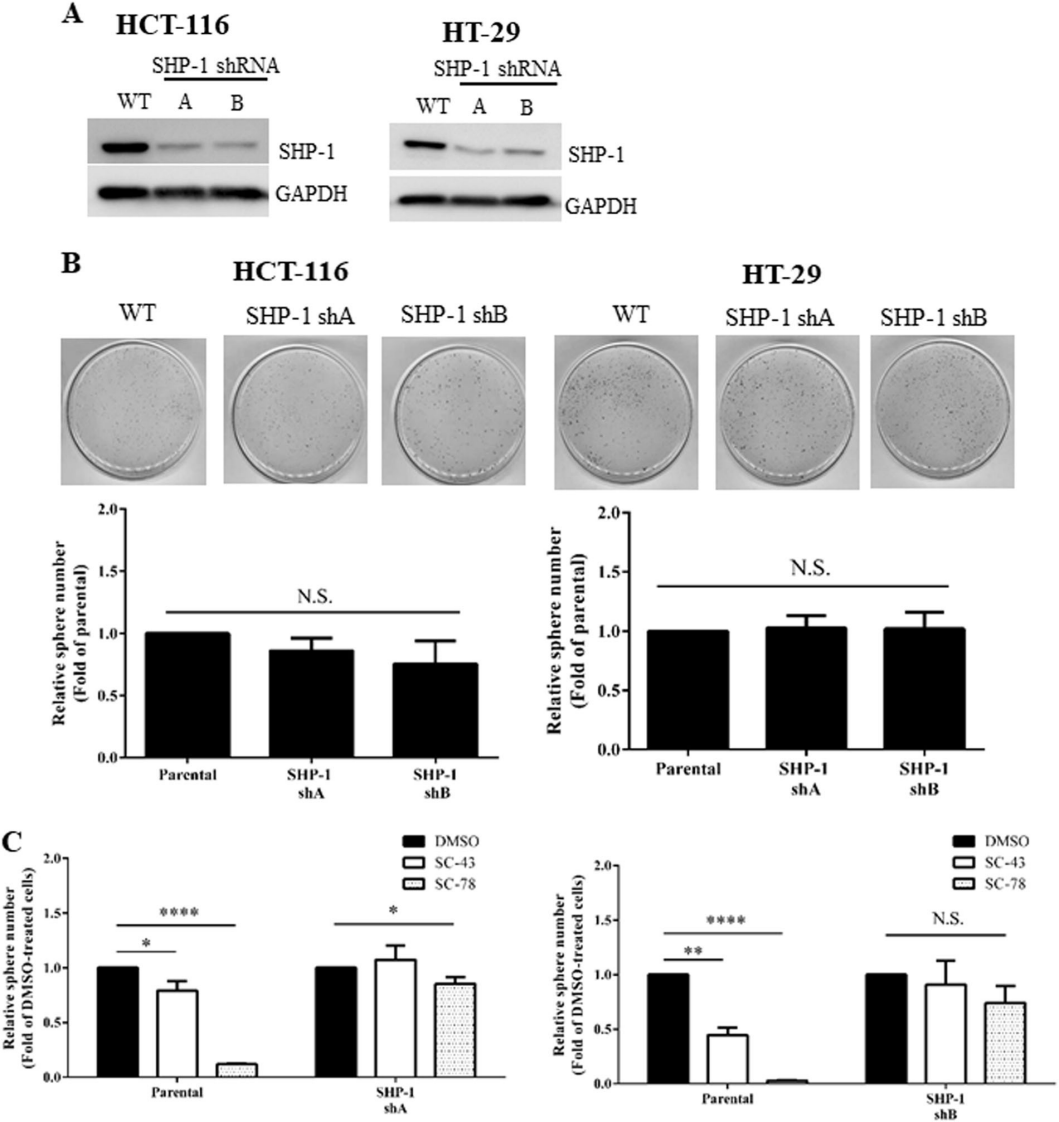
Attenuation of the inhibitory effects of SC-43 and SC-78 on sphere-forming HCT-116 and HT-29 cells by SHP-1 knockdown. **a** Total lysates (20 μg) were prepared from two different SHP-1 knockdown clones (SHP-1 shA and SHP-1 shB) derived from HCT-116 (left) and HT-29 (right) cells and subjected to western blot analyses using an anti-SHP-1 antibody as a probe. GAPDH was used as a loading control. **b** Parental (WT) HCT-116 cells and their SHP-1 knockdown clones (left) as well as parental HT-29 cells and their SHP-1 knockdown clones (right) were cultured in defined media. After 7 days, spheres stained by MTT were photographed (scale bar = 0.7 cm), and their numbers were quantified by MetaMorph software. Data (lower panels) are the mean ± SD of three independent experiments (N.S. indicates no significant difference compared with parental cells by Student’s *t*-test). **c** Parental HCT-116 cells and their SHP-1 knockdown clones (SHP-1 shA) (left) as well as parental HT-29 cells and their SHP-1 knockdown clones (SHP-1 shB) (right) were cultured in defined media containing DMSO (vehicle), 0.5 μM SC-43, or 0.4 μM SC-78 for 7 days. The sphere number was quantified. *p < 0.05, **p < 0.01, ***p < 0.005, and ****p < 0.001 compared with DMSO-treated cells by Student’s *t*-test

To examine whether the suppressive effects of SC-43 and SC-78 on the stemness of human CRC cells could indeed be mediated via SHP-1 activation, we first established SHP-1 knockdown clones from HCT-116 and HT-29 cells by infecting them with lentiviruses expressing two different SHP-1 shRNA constructs. As shown in Fig. 5a, SHP-1 protein levels were markedly reduced in two knockdown clones (SHP-1 shA and SHP-1 shB) derived from HCT-116 and HT-29 cells. We then investigated whether SHP-1 knockdown could affect the sphere-forming ability of the clones. To our surprise, SHP-1 knockdown did not significantly affect the self-renewal ability of these cells (Fig. 5b). On the other hand, except for a small but significant inhibitory effect of SC-78 on the sphere formation of SHP-1 knockdown HCT-116 cells, SC-43 and SC-78 did not interfere with the sphere-forming ability of the remaining SHP-1 knockdown clones (Fig. 5c).

### SC-43 and SC-78 synergize with oxaliplatin and/or irinotecan in suppressing the sphere formation of HCT-116 and HT-29 cells

**Fig. 6:**
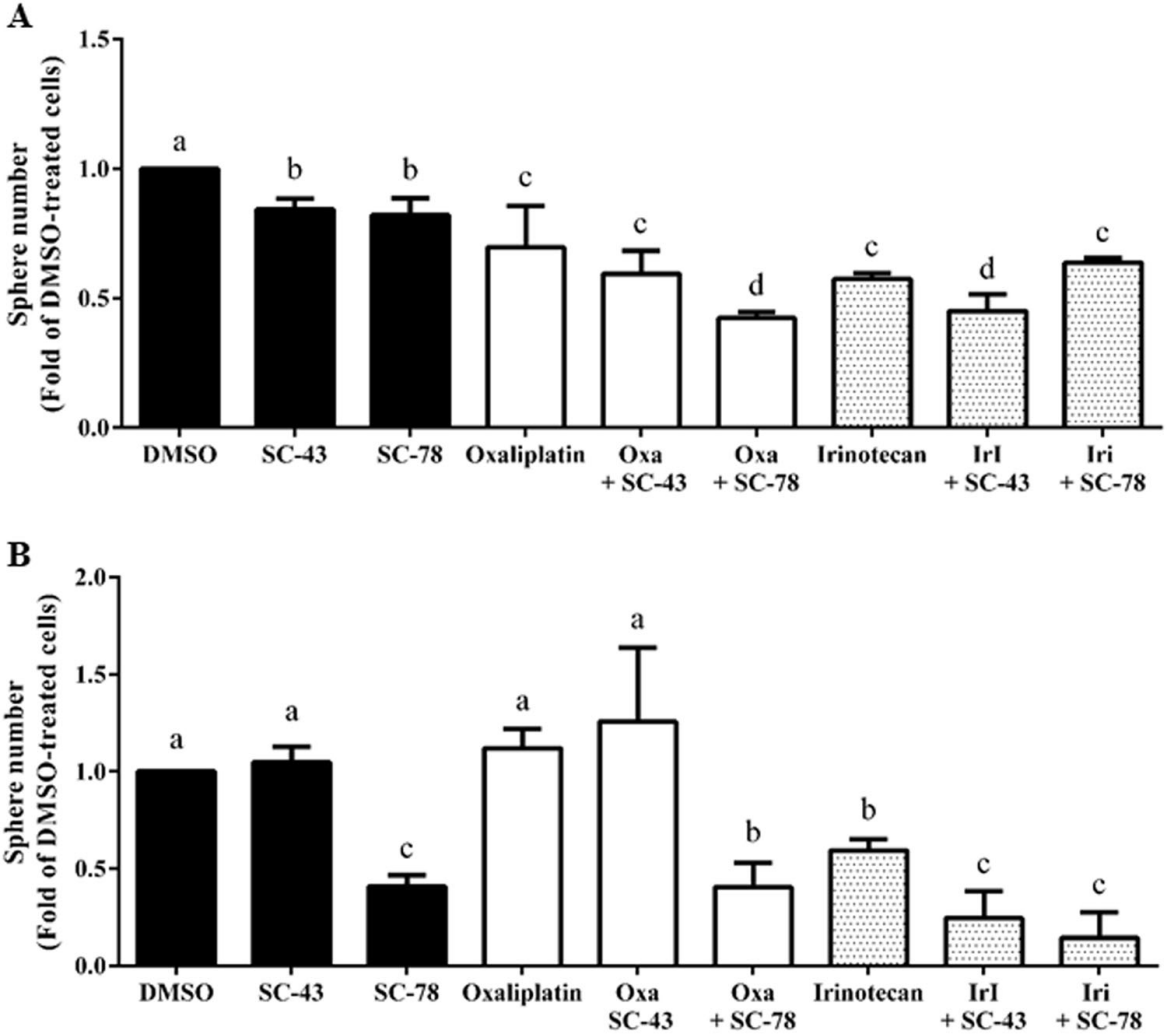
Synergism of SC-43 and SC-78 with oxaliplatin and/or irinotecan in suppressing the sphere formation of HCT-116 and HT-29 cells. **a** HCT-116 and **b** HT-29 cells (1 × 10^5^) were cultured in defined media containing DMSO (vehicle), 0.4 μM SC-43, 0.4 μM SC-78, oxaliplatin (2 μM in HCT-116 and 1 μM in HT-29), 2 μM irinotecan, or different combinations of the four drugs. After 7 days, spheres stained by MTT were photographed, and their numbers were quantified by MetaMorph software. Data (mean ± SD, *N* = 3) were analyzed by one-way ANOVA with the LSD post-hoc test; different letters represent different levels of significance (*p* < 0.05)

We assessed whether SC-43 and SC-78 could synergize with the clinically available anti-CRC drugs in attenuating the stemness of the human CRC cells. Considering that oxaliplatin and irinotecan are the first-line drugs for treating metastatic CRC (mCRC), fixed doses (0.4 μM) of SC-43 and SC-78 were combined with 2 μM oxaliplatin to treat HCT-116 cells, 1 μM oxaliplatin to treat HT-29 cells, and 2 μM irinotecan to treat both cell lines for 7 days before their sphere numbers were counted. The results showed that 0.4 μM SC-43 synergized with 2 μM irinotecan in decreasing the sphere-forming ability of both cell lines, and 0.4 μM SC-78 synergized with 2 μM oxaliplatin in reducing that of only HCT-116 cells (Fig. 6). Together, these results suggest that a combination of these novel SHP-1 agonists with conventional chemotherapeutic agents might be more effective in eradicating stem cell subpopulations in human CRC.

## Discussion

CRC is one of the most prevalent and deadly malignancies worldwide^1^. Currently, no effective treatments are available for patients with advanced cancer, especially the metastatic types. The main challenge in developing successful treatment methods for CRC is the eradication of its stem cell subpopulations (CRCSCs), which are responsible for invasion/metastasis and therapy resistance^6–9^. Among various hyperactive signaling pathways found in CRCSCs, STAT3 is an attractive therapeutic target, and several natural and synthetic small-molecule STAT3 inhibitors have been developed^17–23^. However, napabucasin, whose action mechanism has not been clearly defined, is the only compound that has exhibited efficacy in treating patients with mCRC and metastatic pancreatic adenocarcinoma when combined with other conventional chemotherapeutic agents in clinical studies^24^. Therefore, other novel small-molecule STAT3 inhibitors with a well-defined mechanism of action should be developed.

In this study, we first determined the role of STAT3 signaling in CRCSCs characterized by drug resistance. Elevated pSTAT3 levels were detected in oxaliplatin-resistant HCT-116 and HT-29 cells as well as in irinotecan-resistant HT-29 cells (Fig. 1). Moreover, higher pSTAT3 levels were also observed in the CD133^+^/CD44^+^ stem cell-like subpopulations of both human CRC cell lines (data not shown). These results suggest that activated STAT3 might be a possible therapeutic target in CRC.

Three known STAT3 inhibitors (regorafenib^35^, SC-43^36^, and SC-78^37^) were used to assess the effects of STAT3 inhibition on the stemness properties of the human CRC cells. Regorafenib was more effective than SC-43 and SC-78 in suppressing the growth of HCT-116 cells; however, their inhibitory effects on the growth of HT-29 cells were similar (Table 1). On the other hand, in contrast to SC-43 and SC-78, 4 μM regorafenib failed to reduce the levels of active STAT3 in both cell lines (Fig. 2). In agreement with these findings, the two novel SHP-1 agonists were much more potent than regorafenib in suppressing the sphere formation (Fig. 3a), inducing the sphere shrinkage (Fig. 3b), and inhibiting the soft agar colony formation (Fig. 3c) of both HCT-116 and HT-29 cells. However, there were no differences in the inhibitory effects of the three drugs on the CD133^+^/CD44^+^ stem cell-like subpopulations of both cell lines (Fig. 3d). With respect to the efficacies of the three drugs, SC-43 and SC-78 were more consistent than regorafenib in suppressing the expression of several CRCSC marker genes; the two novel SHP-1 agonists markedly downregulated both the mRNA and protein levels of ALDH1 and Ascl2 in HCT-116 and HT-29 cells, respectively (Fig. 4). Notably, SC-43 and SC-78 but not regorafenib also consistently reduced the mRNA level of Lgr5, which is not only a downstream target of Wnt signaling but also an amplifier of this pathway (Fig. 4c, d). Collectively, these results indicated that SC-43 and SC-78 were much more potent than regorafenib in attenuating the in vitro stemness of the human CRC cells.

To confirm that the suppressive effects of SC-43 and SC-78 on stemness could indeed be mediated by SHP-1 activation, stable SHP-1 knockdown clones were established from HCT-116 and HT-29 cells. As expected, the sphere-forming ability of both cell lines was not affected by SHP-1 knockdown even though more than 50% of the (inactive) enzymes were reduced (Fig. 5a, b). In contrast, the inhibitory effects of both SHP-1 agonists on the sphere formation of the human CRC cells were almost completely abrogated by SHP-1 silencing (Fig. 5c), strongly suggesting that SHP-1 activation is essential for the suppressive effects of SC-43 and SC-78 on stemness. Considering that napabucasin has mostly been clinically used as a small-molecule STAT3 inhibitor with other conventional chemotherapeutic agents^24^, we also assessed the effects of various combinations of SC-43 or SC-78 with two first-line anti-mCRC drugs on the suppression of the self-renewal ability of the human CRC cells. SC-43 exhibited synergistic inhibitory effects on sphere-forming HCT-116 cells with both oxaliplatin and irinotecan, whereas SC-43 exhibited synergistic inhibitory effects on sphere-forming HT-29 cells with only irinotecan (Fig. 6). These findings suggest that further studies are needed to determine suitable combinations of SHP-1 agonists and currently available drugs for in vivo tests.

Chemically, SC-43, SC-78, and regorafenib are structural relatives that contain a core backbone of phenylurea. On the other hand, only regorafenib has kinase inhibitory activity because of its amide-pyridine ring, which forms two hydrogen bonds in the ATP-binding domain of various kinases^44^. In contrast, this is absent in the structure of SC-43 and SC-78, which could exhibit higher target specificity and potency in SHP-1 activation. Overall, the structural differences between regorafenib and the novel agents SC-43 and SC-78 might account for the stronger suppressive effects of the latter two agents on the stemness of human CRC cells. From a drug development point of view, toxicity is a major concern, and molecules with multiple targets are often linked to unwanted toxicity. In this regard, the target-selective SC-43 and SC-78 would more easily fulfill the requirements of clinical trials, which typically aim to minimize toxicity risk. Furthermore, the two-step synthetic procedure of SC-78 and SC-43 is a short synthetic path with high yield, which can reduce the time-consuming process of compound amplification for subsequent preclinical evaluation. Therefore, eradication of the stem cell subpopulations in human CRC by activating SHP-1 with SC-78 and SC-43 may have therapeutic potential and should be further investigated.

## Materials and methods

### Cell cultures and establishment of drug-resistant and SHP-1 knockdown clones

HCT-116 and HT-29 human cancer cell lines purchased from the American Type Culture Collection (ATCC) were maintained in RPMI-1640 medium supplemented with 10% fetal bovine serum (Gibco, USA), 100 units/ml penicillin, 100 μg/ml streptomycin, and 25 μg/ml amphotericin B (1% PSA) (Biological Industries, USA) at 37 °C with 5% CO_2_. To obtain drug-resistant cell lines, HCT-116 and HT-29 cells were cultured with gradually increasing concentrations of oxaliplatin and irinotecan until their growth was no longer affected by these drugs. HCT-116 cells could grow well in the presence of 2 μg/ml oxaliplatin, whereas HT-29 cells could be maintained in media containing 0.5 μg/ml oxaliplatin and irinotecan. To establish SHP-1 knockdown clones from the HCT-116 and HT-29 cell lines, cells were infected with lentiviruses expressing two different shRNA constructs against SHP-1 (SHP-1 shA and SHP-1 shB). The plasmids were purchased from the National RNAi Core Facility (Academia Sinica) and prepared as previously described. Pooled HCT-116 and HT-29 clones were established after the infected cells were cultured in media containing 1.5 and 1 μg/ml of puromycin, respectively, for several weeks.

### Thiazolyl blue tetrazolium bromide (MTT) assay

Cells (1 × 10^4^/well) were seeded into 96-well plates 1 day before treatment with various doses of drugs (regorafenib, SC-43, and SC-78). After 48 h, 1 mg/ml of MTT (Sigma) was added to the wells, and the cells were incubated for another 2 h before the crystals were dissolved by lysis buffer (40 mM HCl in isopropanol). The optical density (OD) of each well was then measured by an ELISA reader (BioRad, USA) at 570 nm. Cells incubated in media containing 0.1% DMSO were used as the untreated vehicle control.

### Western blotting

For total lysate preparation, cells were washed with cold PBS and scraped into 1 ml cold PBS. After centrifugation, the cells were lysed in RIPA buffer [50 mM Tris–HCl, 150 mM NaCl, 0.1% sodium dodecyl sulfate (SDS), and 1% Nonidet P-40 (NP-40); pH 7.4]. Total lysates (20 μg) were separated on a 10% SDS-polyacrylamide gel and processed for immunoblotting with the indicated primary antibodies (Appendix 1). After overnight incubation at 4 °C, the blots were probed with horseradish peroxidase-conjugated secondary antibodies. Signals were detected using an enhanced chemiluminescence system (ECL; NEN Life Science, Boston, MA, USA), and their intensities were quantified by densitometry (ImageJ).

### Sphere formation assay

Sphere formation assay was conducted by seeding 5 × 10^4^ cells onto a Petri dish (Corning) containing serum-free RPMI medium supplemented with N2 (Life Technologies), 20 ng/ml epidermal growth factor (EGF; PeproTech), 10 ng/ml basic fibroblast growth factor (bFGF; PeproTech), 6 mg/ml glucose, 4 mg/ml bovine serum albumin (BSA), and 1% PSA (defined media) for 7 days. To evaluate the effects of regorafenib, SC-43, and SC-78 (alone or combined) on the sphere-forming ability of the cells, the drugs were initially added to the defined media. To assess the effectiveness of the drugs in causing the shrinkage of pre-formed spheres, 1 × 10^5^ cells were maintained in the defined media for 5 days before treatment with the drugs for another 7 days. Spheres were then stained with MTT (1 mg/ml) for 4 h and counted using MetaMorph software (Vanderbilt University’s Institute for Software Integrated Systems, Nashville, TN, USA).

### Soft agar colony formation assay

Cells (5 × 10^3^) resuspended in 4 ml RPMI medium were first mixed with 1 ml of pre-warmed (37 °C) 1.5% agarose (in medium) and incubated in three wells of a 6-well plate (1 ml/well) coated with 1 ml 0.6% agarose (in medium). After 3 weeks of culture, during which the medium was replaced every 3 days, the colonies were stained with MTT and counted as described in the previous section.

### Quantitative RT-PCR (qRT-PCR)

Total RNA was isolated from cells using TRIzol reagent (MDBio, Inc.), and 5 μg of the total RNA was reverse-transcribed using MMLV RT (Fermentas). PCR amplification was carried out with SYBR Green using the CFX Connect™ Real-Time PCR Detection System (Bio-Rad) with various primer sets (Lgr5, F: 5′-TGCTGGCTGGTGTGGSTGCG-3′ and R: 5′-GCCAGCAGGGCAGAGCAA-3′; Ascl2, F: 5′-GTGAAGCTGGTGAACTTGGGC-3′ and R: 5′-CAGCGTCTCCACCTTGCTCA-3′; ALDH1, F: 5′-GCACGC CAGACTTACCTGTC-3′ and R: 5′-CCTCCTCAGTTGCAGGATTAAAG-3′; Bmi1, F: 5′-ACTTCATTGATGCCACAACC-3′ and R: 5′-CAGAAGGATGAGCTGCATAA-3′; GAPDH, F: 5′-GACCACAGTCCATGCCATCAC-3′ and R: 5′-TCCACCACC CTGTTGCTGTAG-3′) designed to analyze the expression of specific genes. The reaction conditions were: 95 °C for 10 min, 40 cycles at 95 °C for 30 s and 65 °C for 30 s, and 72 °C for 30 s. The relative quantity of target gene expression was calculated using the comparative Ct method (ΔΔCt), which was normalized to endogenous GAPDH levels using CFX Manager version 3.1 (BioRad).

### Flow cytometry

Cells treated without and with drugs were detached from the dishes using Tryple^TM^ Express Enzyme (Gibco, USA) and resuspended at 1 × 10^6^ cells/ml in 0.1 ml complete RPMI culture medium containing FITC-labeled CD44 antibody (293C3; Miltenyi Biotec) and PE-labeled CD133 antibody (Beckman Coulter, Inc.) After incubation at 4 °C for 1 h, flow cytometry was performed to identify the CD44^+^ and CD133^+^ subpopulations of each sample (FACSCanto; BD).

## Electronic supplementary material

The chemical structure of (A) regorafenib, (B) SC-43 and (C) SC-78
