# Supplementary figures and images for: Two novel SHP-1 agonists, SC-43 and SC-78, are more potent than regorafenib in suppressing the in vitro stemness of human colorectal cancer cells
Source: Cell Death Discov. 2018 Aug 13;4:82. doi: 10.1038/s41420-018-0084-z (PMC6089896; doi:10.1038/s41420-018-0084-z)

## Slide 1
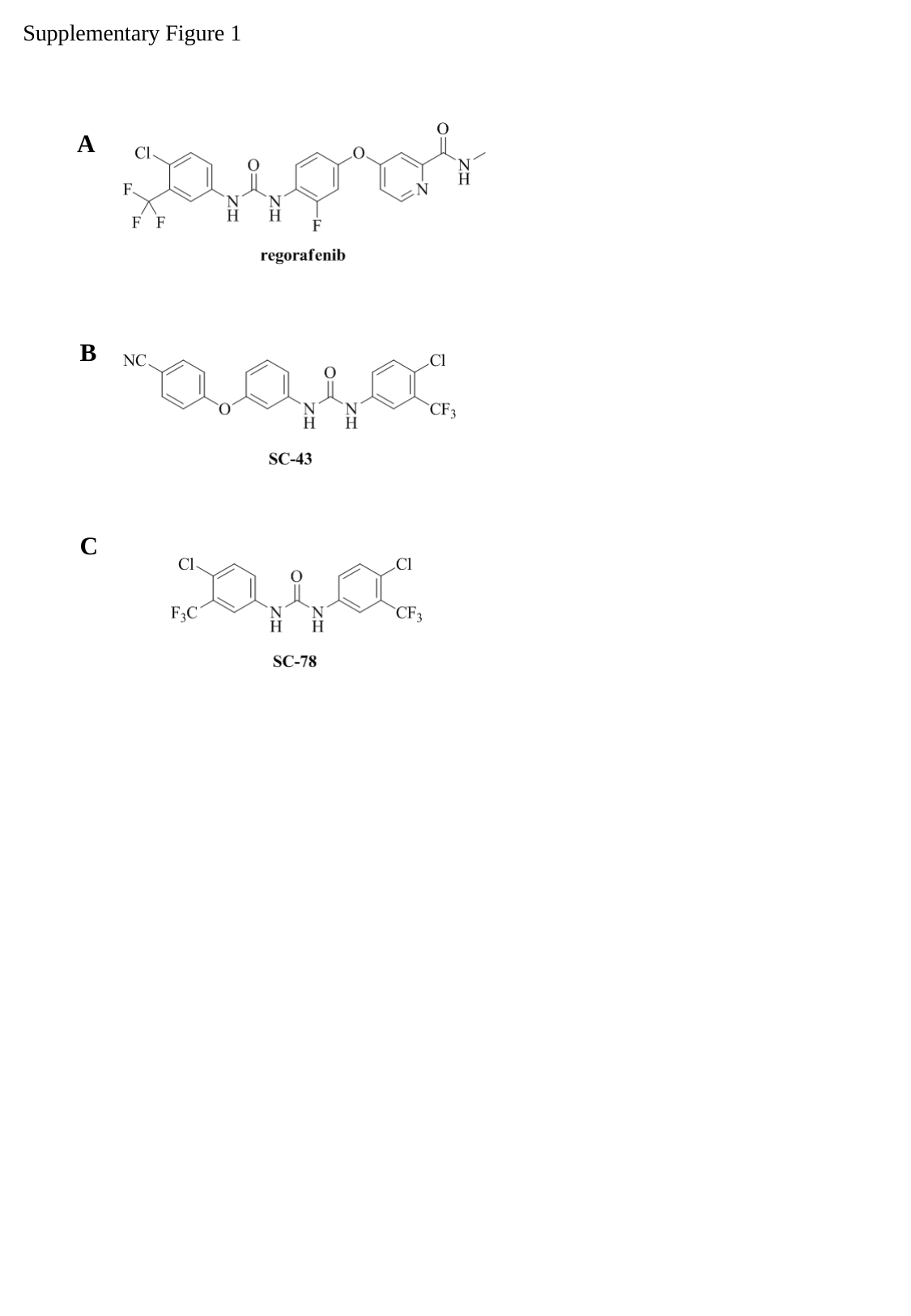

Supplementary Figure 1
A
B
C

Supplement: Supplementary file 1 — The chemical structure of (A) regorafenib, (B) SC-43 and (C) SC-78 [file 41420_2018_84_MOESM1_ESM.pptx]
